# Supplementary material for: Time-series transcriptome analysis identified differentially expressed genes in broiler chicken infected with mixed Eimeria species
Source: Front Genet. 2022 Aug 8;13:886781. doi: 10.3389/fgene.2022.886781 (PMC9393255; doi:10.3389/fgene.2022.886781)
Supplement: Supplementary file 2 [file DataSheet1.ZIP › 4dpi_KEGG.Gsea.1625100298584/KEGG_BETA_ALANINE_METABOLISM.html]

Details for gene set KEGG\_BETA\_ALANINE\_METABOLISM[GSEA]

|  || Dataset | TMM\_4dpi\_gct\_format\_4dpi\_gct\_format.Class\_4dpi.cls #PC\_versus\_NC.Class\_4dpi.cls #PC\_versus\_NC\_repos |
| Phenotype | Class\_4dpi.cls#PC\_versus\_NC\_repos |
| Upregulated in class | 0 |
| GeneSet | KEGG\_BETA\_ALANINE\_METABOLISM |
| Enrichment Score (ES) | -0.54289293 |
| Normalized Enrichment Score (NES) | -1.5095983 |
| Nominal p-value | 0.04688909 |
| FDR q-value | 0.18910135 |
| FWER p-Value | 0.9254 |
Table: GSEA Results Summary

  

Fig 1: Enrichment plot: KEGG\_BETA\_ALANINE\_METABOLISM      
 Profile of the Running ES Score & Positions of GeneSet Members on the Rank Ordered List

  

| SYMBOL | TITLE | RANK IN GENE LIST | RANK METRIC SCORE | RUNNING ES | CORE ENRICHMENT || 1 | CNDP1 | na | 269 | 1.215 | 0.1232 | No |
| 2 | MLYCD | na | 1000 | 0.671 | 0.1427 | No |
| 3 | ALDH3A2 | na | 5110 | 0.069 | -0.1915 | No |
| 4 | AOC3 | na | 5873 | 0.004 | -0.2546 | No |
| 5 | HADHA | na | 7423 | -0.129 | -0.3683 | No |
| 6 | DPYD | na | 7729 | -0.155 | -0.3752 | No |
| 7 | HIBCH | na | 9742 | -0.378 | -0.4975 | Yes |
| 8 | UPB1 | na | 10090 | -0.428 | -0.4752 | Yes |
| 9 | DPYS | na | 10120 | -0.433 | -0.4257 | Yes |
| 10 | ALDH7A1 | na | 10220 | -0.448 | -0.3803 | Yes |
| 11 | SRM | na | 10221 | -0.448 | -0.3266 | Yes |
| 12 | ALDH9A1 | na | 10288 | -0.458 | -0.2772 | Yes |
| 13 | EHHADH | na | 10699 | -0.538 | -0.2469 | Yes |
| 14 | ABAT | na | 11359 | -0.728 | -0.2146 | Yes |
| 15 | ECHS1 | na | 11432 | -0.767 | -0.1288 | Yes |
| 16 | SMS | na | 11937 | -1.476 | 0.0061 | Yes |
Table: GSEA details [plain text format]

  

Fig 2: KEGG\_BETA\_ALANINE\_METABOLISM      
 Blue-Pink O' Gram in the Space of the Analyzed GeneSet

  

Fig 3: KEGG\_BETA\_ALANINE\_METABOLISM: Random ES distribution      
 Gene set null distribution of ES for **KEGG\_BETA\_ALANINE\_METABOLISM**
